# A retrospective study of risk factors for early-onset neonatal sepsis with intrapartum maternal fever

**DOI:** 10.7717/peerj.13834

**Published:** 2022-08-12

**Authors:** Hongmin An, Wei Zheng, Qinghua Zhu, Yun Chai

**Affiliations:** 1 Obstetrics Department, Hangzhou Women’s Hospital, Hangzhou, China; 2 Department of Gastroenterology, Children’s Hospital, Zhejiang University School of Medicine, Hangzhou, China

**Keywords:** Maternal fever, Neonatal sepsis, Chorioamnionitis, White blood cell, Risk factors

## Abstract

**Background:**

Intrapartum fever is a well-known risk factor for adverse perinatal outcomes. In this study, we evaluated the clinical features for intrapartum maternal fever and investigated the risk factors for neonatal early-onset sepsis (EOS) with intrapartum maternal fever.

**Methods:**

This retrospective cohort study involved a total of 568 neonates born to mothers with intrapartum maternal fever (temperature peak ≥38 degree Celsius) in Hangzhou Women’s Hospital from January 1st to December 31st, 2019. Neonates were assigned to the EOS and non-sepsis groups based on the diagnostic criteria for early-onset neonatal sepsis,. Demographic data, clinical information and laboratory test results were evaluated to assess the risk factors for EOS.

**Results:**

A total of 568 neonates were included in this study, 84 of whom were diagnosed with EOS. The EOS group was significantly different from the non-sepsis group in 11 items including the both white blood cell (WBC) count and C-reactive protein (CRP) level of the mother before delivery (*p* < 0.05). A logistic regression analysis revealed that a high maternal WBC count before delivery (OR = 3.261, *p* = 0.019) and a maternal histological chorioamnionitis (HCA) diagnosis (OR = 5.608, *p* = 0.002) were independent risk factors for EOS. The optimal cut-off value for WBC (before delivery) was 16.75 × 10*^9^/L for EOS, according to receiver operating characteristic analysis (area under curve was 0.821).

**Conclusions:**

Elevated prenatal maternal WBC counts and maternal HCA diagnosis are both independently associated with EOS. Prenatal maternal WBC counts can be used as a sensitive indicator to predict EOS early.

## Introduction

Early-onset neonatal sepsis (EOS) is defined as sepsis that manifests within the first 72 h of life and is usually caused by vertical transmission through contaminated amniotic fluid or during vaginal delivery (Stoll et al., 2020; Benitz & Achten, 2021). The most common and characteristic manifestation of neonatal sepsis is a change in feeding behavior in late-onset sepsis and respiratory distress in early-onset sepsis. The baby, who had been active and sucking well, gradually or suddenly becomes lethargic, inactive or unresponsive, and refuses to suckle. Hypothermia is a common manifestation of sepsis, whilst fever is infrequent. Diarrhea, vomiting and abdominal distension may occur. Episodes of apneic spells or gasping may be the only manifestation of septicemia. A critical neonate may develop shock, bleeding and renal failure (Shane, Sánchez & Stoll, 2017). Despite advances in obstetric care, early-onset neonatal sepsis remains a clinical challenge that is associated with high neonatal morbidity and mortality rates (Fleischmann-Struzek et al., 2018; Kim, Polin & Hooven, 2020). In 2018, neonatal sepsis was estimated at a population level of 2,202 cases per 100,000 live births, with a mortality rate of between 11% and 19%. Globally, this represents three million cases of sepsis each year (Fleischmann-Struzek et al., 2018). Given these alarming statistics, it is important to find methods of early diagnosis, investigate potential causes of EOS and develop better treatment options.

Clinical signs of neonatal sepsis range from sub-clinical infection to severe manifestations of focal or systemic disease (Shane, Sánchez & Stoll, 2017). Due to its broad spectrum of clinical manifestations, it is difficult to diagnose early neonatal sepsis. Several researchers have described the risk factors associated with neonatal sepsis (Stoll et al., 2011). These include: delivery at <37 weeks of gestation (Soman, Green & Daling, 1985), maternal Group B Streptococcal (GBS) infection (Stoll et al., 2011), prolonged rupture of membranes ≥ 18 h (Herbst & Källén, 2007), and maternal chorioamnionitis (Yancey et al., 1996). Current research has focused on developing a predictive model for late-onset neonatal sepsis or EOS in full-term and late preterm infants only (Kudawla, Dutta & Narang, 2008; Escobar et al., 2014).

Intrapartum fever is defined as a temperature ≥ 38 degree Celsius (38 °C) during labour or within 24 h after delivery (Ashwal et al., 2018). Previous studies suggest that the rate of intrapartum fever is 3.3%–7% (Wendel et al., 2002; Braun et al., 2016). Peripartum maternal fever of 38 °C or higher is a risk factor for EOS, and if present, requires prophylactic antibiotic treatment for infants, even for clinically healthy newborns (Towers et al., 2017). The US Centers for Disease Control and Prevention (CDC) guidelines state that “in an effort to avert neonatal infections, maternal fever alone in labor may be used as a sign of chorioamnionitis and hence indication for antibiotic treatment” (American College of Obstetricians and Gynecologists, 2011; Committee on Infectious Diseases et al., 2011). Facilities that choose to follow these guidelines need to identify these newborns and begin intravenous antibiotic therapy. Some facilities may be able to treat newborns with intravenous antibiotics in a general neonatal unit where the mother and baby are in the same room, while others may need to transfer the newborn to a neonatal intensive care unit for treatment, which may separate the mother from the newborn. In most hospitals in China, newborns stay with their mothers on the postnatal floor and there is no special nursery for healthy newborns; intravenous antibiotics are often administered in the NICU.

Given the variety of causes of intrapartum maternal fever, its association with neonatal sepsis has not been fully determined (Ashwal et al., 2018). Previous studies have investigated the role of maternal fever as an indicator of neonatal sepsis with inconsistent results (Towers et al., 2017; Ashwal et al., 2018). In 2017, a study by Towers et al. (2017) found that 0.24% of 412 newborns of mothers with intrapartum fever were diagnosed with neonatal sepsis, but less than 0.0007% of the remaining 5,697 newborns of mothers without fever received a neonatal sepsis diagnosis. In contrast, another study found that out of 13,887 live births, 18 full-term newborns developed infections, all of which occurred prior to maternal fever (Wendel et al., 2002). Maternal fever may suggest maternal-fetal transmission of infection leading to neonatal sepsis, but it may also be non-specific. Neonates born to mothers without fever can also develop sepsis, highlighting the uncertainty of the correlation between maternal fever and EOS (Gupta et al., 2021). Therefore, it may not be reasonable to routinely administer antibiotics to all newborns of mothers with fever at delivery. Treating healthy neonates with unneeded antibiotics can be detrimental (Strunk et al., 2018a; Strunk et al., 2018b). Longer hospital stays for tests and treatment may lead to higher medical costs. The infant microbiome is also very sensitive, and exposure to avoidable antibiotics may have long-term health outcomes; prescribing avoidable antibiotics also impacts overall antibiotic stewardship (Gupta et al., 2021).

China has a large population base and a large number of births each year with many newborns suffering from sepsis. Few studies have been reported on intrapartum maternal fever and neonatal sepsis. It is important to study the relationship between maternal fever and neonatal sepsis at the frontline of clinical practice in China. Therefore, the aim of this study was to identify the predictors of EOS. We retrospectively evaluated neonates with febrile mothers during delivery and explored the risk factors of neonatal sepsis to inform the early clinicalassessment and prediction of neonatal sepsis risk.

## Materials and Methods

### Patients and data selection

A retrospective cohort study involving neonates born to mothers with intrapartum fever in Hangzhou Women’s Hospital was performed from January 1st to December 31st, 2019. The medical records of the subjects between 37^0/7^ and 41^0/7^ gestational age who experienced active labor (induced or spontaneous) and experienced a systemic fever of ≥ 38.0 °C along with the records of their respective neonates were reviewed retrospectively. Subjects whose medical records were incomplete, experienced non-singleton gestations, stillbirths, scheduled cesarean sections, had infants delivered prior to 37^0/7^ gestational age, or had congenital fetal anomalies were not included in this analysis.

**Figure 1:**
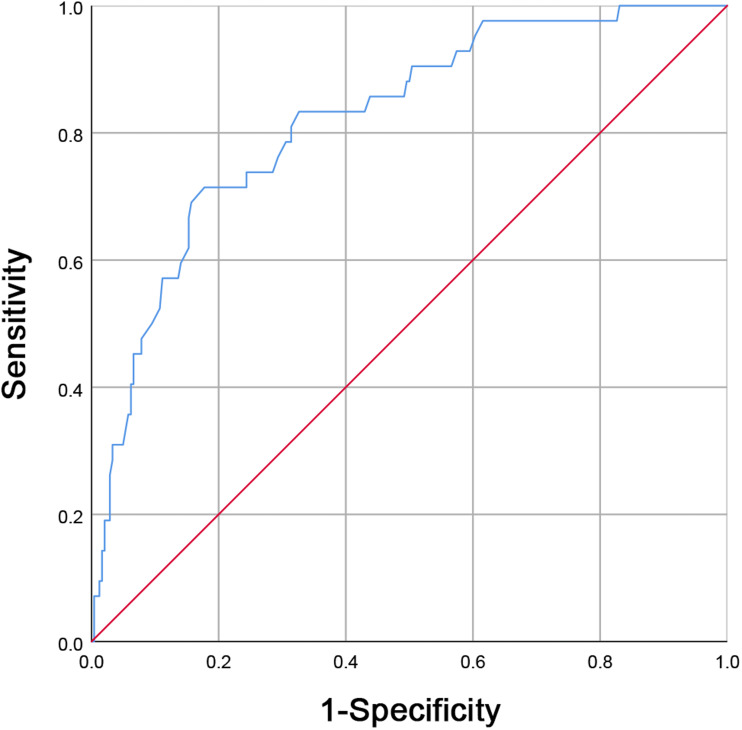
Receiver operating characteristic (ROC) curve analysis for maternal white blood cell (WBC) counts before delivery in predicting neonatal early-onset sepsis (EOS). A maternal WBC level greater than 16.75 × 10*^9^/L (area under the curve was 0.821, with sensitivity and specificity being 71.40% and 82.20%, respectively) is a good predictor for EOS.

The oral temperature of the parturients were measured routinely prior to entering the labor room, as well as every eight hours thereafter. When the oral temperature measured ≥ 37.5 °C, additional temperature measurements followed in the next hour. These procedures are summarized in the flow diagram in Fig. 1. Maternal baseline data, intrapartum manifestations, neonatal outcomes, and obstetric outcomes were acquired through internal reviews of the electronic medical records. This study was approved by the Medical Ethics Committee of Hangzhou Women’s Hospital (2020A10-24). The participants provided written informed consent to participate in this study.

### Data collection of baseline characteristics

The maternal baseline features and intrapartum manifestations collected were: maternal age, parity, gestational age, gender, body mass index (BMI), education, epidural analgesia, white blood cell (WBC) count before delivery, C-reactive protein (CRP) levels before delivery, prothrombin time (PT) before delivery, activated partial thromboplastin time (APTT) before delivery, peak fever temperature, premature rupture of membranes (PROM) ≥ 18 h, meconium-stained amniotic fluid III (MSAF III), the length of the first stage, second stage and third stage of labor, WBC count after delivery, CRP levels after delivery, gestational hypertension, and gestational diabetes mellitus and hypothyroidism, which is linked to intra-uterine temperature in labor (Cheng et al., 2007).

### Diagnosis of neonatal sepsis

Neonatal sepsis was diagnosed by the clinical findings and the presence of a positive blood culture, in combination with clinical and laboratory parameters (Vergnano et al., 2011). The diagnosis of neonatal sepsis in this study was based on the clinical diagnostic criteria from the study “Diagnosis and treatment of neonatal sepsis” (Polin, 2012; Lutsar et al., 2014; Subspecialty Group of Neonatology Pediatrics Society Chinese Medical Association, 2019). Confirming the diagnosis requires clinical manifestations and a positive culture of blood or cerebrospinal fluid (or other sterile cavity fluid) (Kim, Polin & Hooven, 2020).

Clinically diagnosed neonatal sepsis requires clinical manifestations that meetany of the following conditions: I. A positive test for DNA of pathogenic bacteria in blood specimen; II. The result of cerebrospinal fluid examination suggested the diagnosis of suppurative meningitis; III. Blood test results meet at least two of the following criteria: i. WBC: leukopenia (<5 × 10*^9^/L) or leucocytosis (<3 days WBC ≥ 30 × 10*^9^/L, ≥ 3 days WBC ≥ 20 × 10*^9^/L); ii. Immature/total neutrophils (I/T): <3 days I/T ≥ 0.16, ≥ 3 days I/T ≥ 0.12; iii. CRP ≥ 10mg/L; iv. Blood platelet count <100 × 10*^9^/L; v. Serum procalcitonin ≥ 0.5 mg/L. Based on these diagnostic criteria for EOS, neonates were divided into the EOS and non-sepsis group.

### Diagnosis of histological chorioamnionitis

Histological chorioamnionitis (HCA), an inflammation of the fetal membranes, is associated with adverse maternal and neonatal outcomes, including preterm birth, early-onset sepsis and necrotizing enterocolitis (Sweeney et al., 2016; Strunk et al., 2018a; Strunk et al., 2018b). In this study, we used the description of the maternal inflammatory response found in the Amsterdam Placental Workshop Group Consensus Statement to diagnose HCA (Khong et al., 2016). All placental histology was reported by two pathologists specializing in perinatal pathology. The diagnosis of HCA and the assessment of its severity were made according to the Blanc classification: polymorphonuclear lymphocytes infiltrating the subchorionic area (stage I), infiltration of the chorionic membrane (stage II), and infiltration of both the chorionic and amniotic membranes (stage III) (Blanc, 1981).

### Statistical analysis

Statistical analyses were performed using SPSS version 20.0 (IBM, Hangzhou, China). Demographic and clinical characteristics of the study cohort were counted using univariate analysis. T test, Mann–Whitney *U*-test and Fisher’s exact test was used to determine significance, respectively, as needed. By analysing the results of univariate analyses, *p* < 0.05 was used as a threshold for the next step into the logistic regression analysis (univariate logistic regression and multivariate logistic regression). Predictors were entered into a binary logistic regression model in order to count independent associations and calculate the odds ratios (OR) with 95% confidence intervals (CIs). Receiver operating characteristic (ROC) curves were derived and the area under the curve (AUC) was analysed and subsequently analysed in comparison with overall accuracy. All *p*-values were two-tailed, and *p* < 0.05 was considered statistically significant.

## Results

### Patient demographics and clinical data

Eighty-four of the 568 newborns satisfied the EOS diagnostic criteria. There were 56 cases with positive blood cultures with the following results: 31 cases of Group B Streptococcus, 18 cases of Enterococcus faecalis, five cases of Escherichia coli and two case of Lactobacillus acidophilus. The remaining 28 neonates were still clinically diagnosed with EOS because of combined abnormal clinical manifestations and laboratory results before and after admission, despite negative blood cultures. Findings from statistical analyses for maternal demographic characteristics and characteristics before and after delivery on 23 parameters, including maternal age, body mass index (BMI), peak fever temperature, premature rupture of membranes (PROM) ≥ 18 h, HCA levels, WBC count before delivery, between the EOS and non-sepsis groups are shown in Tables 1 and 2. The mean age was 28.60 ± 2.66 years in the EOS group and 28.53 ± 2.85 years in the non-sepsis group, with no significant difference between the two groups (*p* = 0.90). Nine of the maternal parameters, including WBC count before delivery, blood neutrophil count (NC) before delivery, CRP levels before delivery, activated partial thromboplastin time (APTT) before delivery, peak fever temperature, PROM ≥ 18 h, WBC count after delivery, NC after delivery and CRP levels after delivery were significantly different in the EOS group when compared to the non-sepsis group (*p* < 0.05). Women whose babies had EOS had a higher peak fever temperature (*p* = 0.002). Maternal incidence of PROM ≥ 18 h was higher in the EOS than in the non-sepsis group (*p* = 0.016). Women with neonates in the EOS group had higher prenatal WBC levels, NC, CRP levels, and APTT before delivery (*p* < 0.05). They also had higher WBC levels, NC, and CRP levels after delivery (*p* < 0.01).

**Table 1:** Maternal demographic characteristics between EOS and non-sepsis groups by *T* test, and Mann–Whitney *U*-test and Fisher’s exact test.

Characteristic	EOS (*n* = 84)	Non-sepsis (*n* = 484)	*p*-value
Maternal age: (mean ± SD) years	28.60 ± 2.66	28.53 ± 2.85	0.90
Parity			0.99
1	82	466	
≥ 2	2	18	
Gestational week: (mean ± SD)	39.36 ± 1.21	39.28 ± 1.02	0.67
BMI: median (IQR)	27.70 (25.05, 29.01)	26.68 (25.00, 28.37)	0.97
Education level			0.48
Master and Doctor	12	62	
University graduate	70	386	
Technical secondary school	2	36	
Epidural analgesia	56	362	0.27

**Notes.**

EOS neonatal early-onset sepsis IQR interquartile range BMI body mass index

**Table 2:** Maternal characteristics before and after delivery between EOS and non-sepsis groups by *T* test, and Mann–Whitney *U*-test and Fisher’s exact test.

Characteristic	EOS (*n* = 84)	Non-sepsis (*n* = 484)	*p*-value
WBC before delivery: (mean ± SD) (×10^*^^9^/L)			
	18.20 ± 4.59	12.45 ± 5.31	0.002^**^
NC before delivery: (mean ± SD) (×10^*^^9^/L)			
	13.32 ± 4.56	10.30 ± 4.38	<0.0001^**^
CRP before delivery: median (IQR), mg/L			
	16.83 (7.56, 34.48)	8.91 (3.37, 20.05)	0.0003^**^
PT before delivery: median (IQR), second			
	10.55 (10.10, 11.03)	10.55 (10.10, 11.00)	0.71
APTT before delivery: median (IQR), second			
	26.80 (24.58, 28.43)	25.70 (24.40, 27.10)	0.04^*^
Peak fever temperature:median (IQR), °C			
	38.70 (38.50, 38.83)	38.40 (38.20, 38.70)	0.002^**^
PROM ≥ 18 h	32	102	0.016^*^
Meconium-stained amniotic fluid III	22	90	0.25
Frist stage of labor, min			
(mean ± SD)	698.32 ± 254.08	635.97 ± 327.78	0.36
Second stage of labor, min			
(mean ± SD)	99.95 ± 60.12	92.06 ± 54.75	0.40
Third stage of labor, min			
(mean ± SD)	10.36 ± 5.88	9.33 ± 5.21	0.25
Gestational hypertension	2	22	0.52
Gestational diabetes mellitus	6	56	0.40
Hypothyroidism	10	78	0.49
WBC after delivery: (mean ± SD) (×10^*^^9^/L)			
	14.20 ± 4.26	12.66 ± 2.91	0.004^**^
NC after delivery: (mean ± SD) (×10^*^^9^/L)			
	11.70 ± 4.04	10.12 ± 2.73	0.002^**^
CRP after delivery: median (IQR), mg/L			
	85.98 (61.71, 132.60)	67.25 (42.53, 97.55)	0.0005^**^

**Notes.**

EOS neonatal early-onset sepsis IQR interquartile range WBC white blood cell count NC neutrophil count CRP C-reactive protein PT prothrombin time APTT activated partial thromboplastin time PROM premature rupture of membranes

* *p* < 0.05

** *p* < 0.01

### Obstetrical and neonatal outcomes in different groups

Findings from the statistical analyses for obstetrical and neonatal outcomes on seven parameters, including assisted vaginal delivery, primary Caesarean section, and maternal HCA diagnosis between the EOS and non-sepsis groups are shown in Table 3. There were 50 male and 34 female newborns in the EOS group and 242 male and 242 female newborns in the non-sepsis group, with no statistically significant difference in gender between the two groups (*p* = 0.32). Two of the parameters—maternal HCA diagnosis and NICU admission—were significantly different in the EOS group when compared to the non-sepsis group (*p* < 0.05). The incidence of maternal HCA was higher in the EOS group than in the non-sepsis group (*p* = 0.0009). Similarly, neonates with EOS had higher rates of NICU admission than neonates born to nonfebrile mothers (*p* < 0.0001). Similar rates of assisted vaginal delivery (*p* = 0.33), primary caesarean section (*p* = 0.14) and low 1-min Apgar scores (*p* = 0.66) were observed between the two groups. Genders and birth weights of the neonates also did not differ between the two groups.

**Table 3:** Obstetrical and neonatal outcomes of the study groups by *T* test, and Mann–Whitney *U*-test and Fisher’s exact test.

Characteristic	EOS (*n* = 84)	Non-sepsis (*n* = 484)	*p*-value
Assisted vaginal delivery	10 (11.90%)	36 (7.44%)	0.33
Primary Caesarean section	28 (33.33%)	110 (22.73%)	0.14
HCA	40	112	0.0009^**^
HCA1	17	63	
HCA2	10	30	
HCA3	13	19	
Newborn gender (Male)	50	242	0.32
Birth weight: median (IQR) g	3420 (3213, 3713)	3425 (3228, 3650)	0.97
1-min Apgar <7	2	18	0.66
NICU admission	84	86	<0.0001^**^

**Notes.**

EOS neonatal early-onset sepsis IQR interquartile range HCA histological chorioamnionitis NICU neonatal intensive care unit

** *p* < 0.01

### Risk factors for EOS

Based on univariate analyses, *p* < 0.05 was set as the threshold for inclusion in logistic regression analyses. Ten maternal parameters were included in this analysis. CRP levels, WBC counts, NC, and APTT before delivery, HCA diagnosis, peak fever temperature, PROM ≥ 18 h, and CRP levels, WBC count, and NC after delivery.A logistic regression analysis (Table 4) confirmed that high maternal WBC counts before delivery (OR = 3.261, 95% CI [1.794–6.318], *p* = 0.019) and a maternal HCA diagnosis (OR = 5.608, 95% CI [1.913–16.436], *p* = 0.002) were both independent risk factors for EOS. Compared to the non-sepsis group, the EOS group exhibited higher prenatal WBC values and a higher incidence of maternal HCA.

**Table 4:** Risk factors for EOS by logistic regression analysis.

Parameters	*p*-value	OR (95% CI)
CRP before delivery	0.058	0.986 (0.973, 1.000)
WBC before delivery	0.019^*^	3.261 (1.794, 6.318)
NC before delivery	0.525	0.920 (0.711, 1.190)
APTT before delivery	0.062	0.886 (0.800, 0.982)
HCA	0.002^*^	5.608 (1.913, 16.436)
Peak fever temperature	0.065	0.570 (0.314, 1.035)
PROM ≥ 18 h	0.079	0.340 (0.111, 1.040)
CRP after delivery	0.522	0.998 (0.991, 1.005)
WBC after delivery	0.530	1.143 (0.754, 1.732)
NC after delivery	0.346	0.807 (0.516, 1.261)

**Notes.**

* *p* < 0.05

EOS neonatal early-onset sepsis OR odds ratio CI confidence interval CRP C-reactive protein WBC white blood cell count NC blood neutrophil count HCA histological chorioamnionitis PROM premature rupture of membranes

### Multivariate logistic regression analyses

By controlling for CRP levels, WBC counts, and APTT before delivery, HCA diagnosis, and peak fever temperature, a multivariate logistic regression analysis showed that maternal WBC levels before delivery was independently associated with EOS risk (OR = 1.582, 95% CI [1.120–2.235], *p* = 0.009) (Table 5).

**Table 5:** Relationship between WBC before delivery and EOS by multiple logistic regression analysis.

Parameters	*p*-Value	AOR (95% CI)
CRP before delivery	0.103	0.990 (0.979, 1.002)
WBC before delivery	0.009^*^	1.582 (1.120, 2.235)
APTT before delivery	0.104	0.929 (0.856, 1.007)
HCA	0.074	0.924 (0.840, 1.016)
Peak fever temperature	0.052	0.463 (0.285, 0.753)

**Notes.**

* *p* < 0.05

WBC white blood cell count HCA histological chorioamnionitis AOR adjusted odds ratio CI confidence interval CRP C-reactive protein APTT activated partial thromboplastin time

### Maternal WBC levels before delivery can predict EOS

With maternal WBC levels before delivery being shown to be independently associated with EOS incidences, we aimed to further establish the value of maternal WBC counts before delivery in EOS prediction. Maternal WBC levels before delivery for 84 patients with EOS and 484 neonates without EOS were compared. Then, an optimal cut-off value for WBC counts before delivery was established by a ROC curve analysis. A maternal WBC level of 16.75 × 10*^9^/L before delivery, with a sensitivity and specificity of 71.40% and 82.20%, respectively, (AUC = 0.821; Fig. 1) was found to be a good predictor for EOS.

## Discussion

In this study, the overall incidence of fever of more than 38 °C was 4.93% (592/12000), which is similar to previous studies (Towers et al., 2017). The overall incidence of fever in mothers of newborns with culture-proven sepsis was 9.86% (56/568), which is much lower than other published reports (Wortham et al., 2016; Gupta et al., 2021), likely due to non-infectious maternal fevers. This is supported by previous studies, which have reported that 40% of women with intrapartum fevers had non-infectious fevers (Kim et al., 2015). Maternal temperature increases as a result of inflammation and may also be influenced by various factors such as the activity and intensity of uterine contractions, as well as physiological changes that occur during labor such as an increase in basal metabolic rates (Anim-Somuah et al., 2018). Other causes of intrapartum fever include antepartum infections, such as urinary/respiratory tract infections, and side effects from medications taken during labor (Gupta et al., 2021). Antepartum and intrapartum characteristics provide important information regarding exposure to infectious diseases and inform neonatologists of obstetric risk factors for neonatal infections (American College of Obstetricians and Gynecologists’ Task Force on Neonatal Encephalopathy, 2014).

Intrapartum fever is monitored to prevent the transfer of infections to newborns with immature immune systems (Gupta et al., 2021). We evaluated the differences in maternal characteristics before and after delivery as well as the obstetrical and neonatal outcomes between the EOS and non-sepsis groups in this study. The results showed that neonates with EOS had mothers with higher peak fever temperatures, higher prenatal WBC levels, and higher CRP levels before delivery. Neonates with EOS born to mothers with intrapartum fevers were also more likely have higher rates of NICU admission than neonates with EOS born to nonfebrile mothers, and mothers with intrapartum fevers whose neonates had EOS had a higher incidence of HCA. This finding indicates that maternal infection plays a role in neonatal EOS. We investigated the risk factors for EOS in neonates born to febrile mothers and evaluated their associations. The results indicated that prenatal maternal WBC elevation and a maternal pathological diagnosis of chorioamnionitis were found to be independent risk factors for neonatal EOS. Our results differ from previous studies that found that the duration of ruptured membranes ≥ 18 h and epidural analgesia were risk factors for maternal fever and neonatal sepsis (Wassen et al., 2014). Randis et al. (2018) reported that babies born to mothers with amnionitis were almost three times more likely to be diagnosed with suspected sepsis compared to those born to mothers who had not been exposed. Our findings are in line with this conclusion. Globally, clinical chorioamnionitis is the most common infection-associated complication in delivery units, affecting 1–6% of pregnancies in the United States (Perry, Rossi & De Franco, 2019; Venkatesh et al., 2019a; Venkatesh et al., 2019b). This syndrome is a risk factor for adverse maternal outcomes, including postpartum hemorrhage followed by uterine atony and sepsis (Chebbo et al., 2016). Newborns born to mothers diagnosed with clinical chorioamnionitis have a higher risk of sepsis (Gowda et al., 2017; Randis et al., 2018), admission to the neonatal intensive care unit (Venkatesh et al., 2019a; Venkatesh et al., 2019b), and neonatal death compared to neonates born to women without this syndrome (Conde-Agudelo et al., 2020; Racusin et al., 2021).

A multivariate logistic regression analysis revealed that maternal WBC levels before delivery are independently associated with EOS risk, suggesting that elevated prenatal white blood cell counts in mothers indicates an increased risk of EOS. WBC count is widely used in clinical practice. Clinically, it is very important to determine the appropriate maternal WBC count cut-off point for early identification of neonatal EOS. Further WBC investigations using an ROC curve analysis in the neonatal EOS group and non-sepsis group revealed a cut-off value of maternal WBC to be 16.75 × 10*^9^/L with a 71.40% sensitivity and an 82.20%. Maternal leukocytosis (WBC ≥ 15.00 × 10*^9^/L) is one of the diagnostic criteria for clinical chorioamnionitis (Gibbs et al., 1982; Yoder et al., 1983). Our results suggest that a maternal WBC ≥ 16.75 × 10*^9^/L is not only indicative of chorioamnionitis but also increases the possibility of neonatal EOS. Using objective clinical data available at birth, Ryan et al. (2019) found that maternal CRP >10 mg/L was associated with a fetal inflammatory response and increased risk of EOS. Puopolo et al. designed a predictive model for EOS for infants born at ≥ 34 weeks gestation, they found that the two most important predictors were gestational age and maternal fever (American College of Obstetricians and Gynecologists’ Task Force on Neonatal Encephalopathy, 2014). Their model performed well with an AUC of 0.807, which was similar to the AUC of our study.

Our study has several strengths. First, this is one of the few studies in China to evaluate risk factors for early-onset neonatal sepsis with intrapartum maternal fever. Secondly, this was a single-centre cohort study in which all women and newborns received uniform diagnostic criteria and clinical management strategies and all patients with fever underwent placental pathology, which ensured the integrity of the retrospective cohort.

This study has its limitation. As the data collection is retrospective, there is a potential risk of information bias. In order to curtail this potential bias, cases were meticulously screened and reviewed. In addition, this study relied on blood cultures to demonstrate sepsis. However, although blood cultures are the gold standard, they are less sensitive due to the use of antibiotics at delivery and the limitations of blood volume per culture. Therefore, some children with negative blood cultures were still clinically diagnosed with neonatal sepsis based on clinical presentation and other laboratory findings. Since all cases were selected from Hangzhou Women’s Hospital, there is a possibility of results having confounding variables that are inevitable in all single-centered studies. Therefore, large-scale multicenter prospective clinical studies are recommended to confirm our findings. In addition, the practical and clinical significance of the findings is not entirely clear and needs to be further confirmed in future multicentre studies.

## Conclusions

In conclusion, we found that high maternal WBC counts before delivery and maternal HCA diagnosis are both independently risk factors for EOS and that maternal WBC levels before delivery was independently associated with EOS risk. ROC curve analysis showed that a maternal WBC level of 16.75 × 10*^9^/L before delivery, with a sensitivity and specificity of 71.40% and 82.20%, respectively, was a good predictor for EOS. Prenatal maternal WBC counts elevation can be used as a sensitive indicator for the early prediction of EOS, providing an important reference for neonatal pediatricians.

##  Supplemental Information

10.7717/peerj.13834/supp-1 Data S1 Demographic characteristics of all patients

## Additional Information and Declarations

### Data Availability

The following information was supplied regarding data availability: The raw measurements are available in the Supplemental File.
